# Identification of Three Novel *MAGED2* Variants Causing Antenatal Bartter Syndrome in Three Chinese Families

**DOI:** 10.3390/genes17040424

**Published:** 2026-04-03

**Authors:** Shufa Yang, Xiaojuan Li, Haili Jiang, Jiahui Cheng, Changlong Li, Xinyi Xie, Xiaoqin Xiao

**Affiliations:** 1 Prenatal Diagnostic Center, Beijing Obstetrics and Gynecology Hospital, Capital Medical University, Beijing Maternal and Child Health Care Hospital, Beijing 100026, China; sfyang@mail.ccmu.edu.cn (S.Y.); jianghaili2020@ccmu.edu.cn (H.J.); 2 Center for Cellular and Molecular Diagnostics, Sun Yat-sen Memorial Hospital, Sun Yat-sen University, Guangzhou 510655, China; lixj75@mail.sysu.edu.cn; 3 Department of Genetics and Developmental Biology, Capital Medical University, Beijing 100069, China; 18813053310@163.com (J.C.); licl@ccmu.edu.cn (C.L.); 4 Department of Neonatology, The Sixth Affiliated Hospital, Sun Yat-sen University, Guangzhou 510655, China

**Keywords:** transient antenatal Bartter syndrome, *MAGED2*, polyhydramnios, large for gestational age, prenatal diagnosis

## Abstract

**Background/Objectives**: We aimed to report three novel *MAGED2* variants associated with transient antenatal Bartter syndrome (TABS) and to summarize the prenatal and postnatal features of *MAGED2*-related TABS through case analysis and literature review. **Methods**: Three unrelated Chinese families with polyhydramnios-affected pregnancies underwent genetic testing. Clinical data, including prenatal imaging, delivery details, and postnatal outcomes were reviewed. A literature review of reported *MAGED2* variants and associated phenotypes was conducted. **Results**: Three previously unreported *MAGED2* variants were identified: two frameshift variants (c.1511del [p.Gly504Alafs*72] and c.338del [p.Pro113ArgfsTer4]) and one deletion (chrX:54,820,664–54,839,053 [GRCh37]). All fetuses presented with polyhydramnios; two were large for gestational age (LGA). Additional findings included ventriculomegaly and scrotal enlargement. Two male infants were delivered at 33 weeks following repeated amnioreduction, with transient postnatal electrolyte abnormalities and normal neurodevelopment at 3 and 4 years. One fetus with a frameshift variant died in utero at 26 + 1 weeks. A literature review of 53 cases revealed 38 distinct *MAGED2* variants, predominantly null variants (65.8%). Polyhydramnios was the most consistent antenatal sign. No intellectual disability was reported in surviving individuals. **Conclusions**: These findings expand the *MAGED2* mutational spectrum. Polyhydramnios and LGA represents the most frequent features in TABS. In fetuses presenting with early-onset severe polyhydramnios (around 19–20 weeks of gestation), particular attention should be paid to possible exon-level or partial deletions involving *MAGED2* during genetic evaluation.

## 1. Introduction

Bartter syndrome is a group of rare inherited renal tubular disorders characterized by renal salt wasting, polyuria, and secondary hyperaldosteronism, typically accompanied by hypokalemia, low blood pressure, and metabolic alkalosis. To date, pathogenic variants in several genes have been implicated in Bartter syndrome, including *SLC12A1* (MIM*600839), *KCNJ1* (MIM*600359), *CLCNKB* (MIM*602023), *BSND* (MIM*606412), *CLCNKA* (MIM*602024), and *MAGED2* (MIM*300470). Among these, *MAGED2*, located at Xp11.21, is responsible for antenatal, transient Bartter syndrome type 5 (MIM#300971). Bartter syndrome caused by *MAGED2* variants is also referred to as transient antenatal Bartter syndrome (TABS).

*MAGED2* is expressed in the thick ascending limb (TAL) of the loop of Henle and the distal convoluted tubule in humans [1]. TAL plays a pivotal role in the urinary concentrating mechanism of the kidney. Defective salt transport in the TAL represents the central pathophysiological mechanism of Bartter syndrome. The apical Na^+^–K^+^–2Cl^−^ cotransporter, NKCC2, is the principal mediator of sodium chloride reabsorption in TAL epithelial cells [2]. This transcellular transport establishes a lumen-positive transepithelial potential, which is crucial for the paracellular reabsorption of cations, including sodium, potassium, calcium, and magnesium [3,4]. Disruptions in TAL function, whether due to defects in NKCC2 activity or impaired claudin-mediated paracellular transport, lead to significant salt wasting and electrolyte imbalances, characteristic of Bartter syndrome [5,6]. Under hypoxic conditions resembling the intrauterine environment, MAGED2 inhibits MDM2-mediated endocytosis of Gαs, thereby maintaining cAMP production. Elevated intracellular cAMP activates the PKA signaling pathway, which in turn stimulates the NCC and NKCC2 [7,8,9]. Deficiency of MAGED2 disrupts the function of NCC and NKCC2, leading to impaired renal salt transport. In the studies by Radi et al. and Reusch et al., HEK293 cells were used to analyze the mechanistic link between MAGED2, Gαs stabilization, and cAMP/PKA signaling [8,9]. In addition, MAGED2 stabilizes HIF-1α via the Gαs–cAMP/PKA signaling pathway, thereby facilitating cellular adaptation to hypoxic stress [10].These functional characteristics of MAGED2 provide a mechanistic explanation for the transient nature of *MAGED2*-related Bartter syndrome, as polyuria and electrolyte disturbances gradually improve and resolve after birth with the alleviation of hypoxic conditions. Buffet et al. excluded the involvement of differential expression or methylation of the *MAGED2* gene as a potential pathogenic mechanism for TABS by conducting comprehensive analyses, including single-cell RNA sequencing of kidney tissues and methylation profiling of the *MAGED2* promoter region [11].

TABS was first reported in 2016 in nine families, with polyhydramnios identified as the predominant prenatal manifestation [1]. Subsequent reports have expanded the prenatal phenotypic spectrum beyond polyhydramnios. Legrand et al. described fetal macrosomia [12]. Yang et al. reported echogenic foci in the left ventricle and intestine [13]. Walsh et al. observed fetal macrosomia and suspected urethral abnormalities [14]. Buffet et al. reported additional congenital anomalies, including oesophageal atresia, tetralogy of Fallot, microretrognathia, low-set ears, and suspected cleft palate [11]. Postnatally, transient polyuria remains the hallmark clinical feature, often accompanied by varying degrees of dehydration, hyponatremia, hypokalemia, hypochloremia, and metabolic alkalosis [1,12,15]. Elevated plasma renin and aldosterone levels have been reported in a limited number of cases [15].

However, the expanding phenotypic variability, the limited number of reported cases, incomplete characterization of its prenatal clinical features and the identification of exon-level deletions highlight the need for continued refinement of the clinical and molecular spectrum of TABS. In the present study, we report the prenatal findings, perinatal management, and postnatal follow-up of three families with TABS. In addition, by reviewing previously published cases, we comprehensively analyze the prenatal characteristics, therapeutic strategies, and clinical outcomes of TABS.

## 2. Materials and Methods

### 2.1. Study Subjects

A retrospective analysis was conducted on pregnancies managed at Sun Yat-sen Memorial Hospital, Sun Yat-sen University, involving pregnant women presenting with polyhydramnios or a history of polyhydramnios in previous pregnancies. The inclusion criteria include: (1) the presence of pathogenic variants in *MAGED2*; and (2) the occurrence of polyhydramnios during the course of pregnancy. Polyhydramnios was defined as an amniotic fluid index (AFI) ≥ 24 cm (or a deepest vertical pocket ≥ 8 cm).

### 2.2. Genetic Testing

Genetic investigations included chromosome analysis, chromosome microarray analysis (CMA), exome sequencing (ES) or Trio-ES, and genome sequencing (GS).

ES or trio-ES was performed following standard procedures, including library preparation, exome capture, sequencing, bioinformatic analysis, and variant interpretation. Sequencing libraries and target enrichment was performed with llumina DNA Prep with Exome Enrichment Tagmentation Kit (Illumina, San Diego, CA, USA). Bioinformatic analysis was conducted using fastp (v0.23.2), BWA (v0.7.17), GATK (v4.2.6.1), and ANNOVAR (v20220216) for data cleaning, alignment to the hg19 reference genome, variant calling, and variant annotation. The sequencing data were subjected to quality control according to the following criteria: (1) an average read depth of ≥100× across the targeted exonic regions; and (2) the proportion of bases within the exonic regions covered at ≥1×, ≥20×, and ≥30× exceeding 99%, 95%, and 90%, respectively. Variant interpretation was performed according to ACMG variant classification guidelines and supplemental guidelines, combined with clinical manifestations and examination results [16]. CNV analysis in ES/Trio-ES was performed using a read depth (RD)-based algorithm, with a detection limit of copy number changes involving at least two consecutive exons. Detected small variants (<50 bp) were validated by Sanger sequencing, whereas larger variants were confirmed using quantitative PCR, GS, or CMA. In this study, trio-ES was performed in Case 2, while singleton ES was applied in Cases 1 and 3.

GS mainly included library preparation, sequencing, and data analysis. Libraries were constructed using the Illumina DNA PCR-Free Library Preparation Kit (Illumina, San Diego, CA, USA), and sequencing was performed on the Illumina NovaSeq 6000 (Illumina, San Diego, CA, USA). After sequencing, raw data were processed using fastp for data cleaning, followed by alignment to the reference genome using BWA-MEM. The aligned data were subsequently visualized using the Integrative Genomics Viewer (v2.15.6).

CMA was performed using the CytoScan 750K array (Affymetrix, Santa Clara, CA, USA). The main experimental procedures included DNA extraction, DNA digestion, DNA labeling, DNA hybridization, and array scanning. Microarray data were analyzed using the Affymetrix Chromosome Analysis Suite (ChAS) (Affymetrix, Santa Clara, CA, USA), which enables the detection of CNVs, uniparental disomy, and mosaicism.

Chromosome analysis of amniotic fluid mainly involved three steps: cell culture, chromosome preparation, and karyotype analysis. Chromosome preparation included digestion, hypotonic treatment, fixation, slide preparation, aging, and staining to obtain metaphase chromosome spreads. Microscopic analysis was performed according to the International System for Human Cytogenomic Nomenclature (ISCN 2020). A total of 25 metaphase cells were initially analyzed. If mosaicism was suspected, the number of analyzed cells was increased to 100 to determine the proportion of mosaicism.

### 2.3. Breakpoint Junction Analysis by Sanger Sequencing

For the case harboring a deletion involving exons 1–7 of the *MAGED2* gene, breakpoint junction analysis was performed to precisely delineate the genomic breakpoints. Specific primers were designed flanking the predicted breakpoint regions (forward primer: 5′-TGGATCAGAGCTGGCCTTTG-3′; reverse primer: 5′-AGAAGCACCATGAGCAGACC-3′). The upstream primer was located at positions 54,793,900–54,793,919 on chromosome X (NC_000023.11), while the downstream primer was located at positions 54,812,967–54,812,986. Only the genomic allele harboring the deletion could be amplified, yielding a PCR product of 703 bp, whereas no amplification was observed for the wild-type allele. The PCR products were subsequently subjected to Sanger sequencing for breakpoint confirmation.

### 2.4. Literature Review

To analyze the pathogenic characteristics associated with *MAGED2*, a literature search was conducted in the databases PubMed (https://pubmed.ncbi.nlm.nih.gov/), Google Scholar (https://scholar.google.com/), CNKI (https://en.oversea.cnki.net/), and Wanfang (http://g.wanfangdata.com.hk/). The search terms included *MAGED2*, Bartter syndrome, and polyhydramnios. This narrative review synthesizes documented cases from the existing literature, with a comprehensive search conducted through 1 December 2025. In total, 53 affected individuals, including those reported in the present study, were collected and analyzed (Table S2).

### 2.5. Ethics Approval

This study was reviewed and approved in advance by the Ethics Committee of Sun Yat-sen Memorial Hospital, Sun Yat-sen University (approval date: 26 November 2025, approval number: 2025-KY-029-01). All procedures involving human participants adhered to the Declaration of Helsinki 1964 and its subsequent revisions, or other applicable ethical standards.

### 2.6. Consent to Participate and to Publish

All participants in this study were thoroughly informed about the study’s purpose, procedures, potential risks, and benefits. They were assured of their right to withdraw from the study at any time without facing any consequences. Written informed consent was obtained from all patients or their legal representatives, covering both genetic testing and the publication of the findings.

## 3. Results

### 3.1. Clinical and Genetic Characteristics of the Three Cases

A total of three cases were identified in this study. Genetic analysis revealed that all three pregnant women carried previously unreported variants in *MAGED2* (NM_177433.1). Across the three families, four fetuses were identified as carrying pathogenic *MAGED2* variants. All affected fetuses developed polyhydramnios. For one fetus, additional clinical information was unavailable. Among the three fetuses with complete clinical data, polyhydramnios was first detected at 20–21 weeks of gestation (median, 20 w), with AFI ranging from 19.1 to 44.8 cm. Two of these three fetuses also presented with LGA, central atrial septal defect, and bilateral testicular hydrocele. The detailed clinical features of each case are described below.

#### 3.1.1. Case 1: c.1511del (p.Gly504Alafs*72)

**Figure 1:**
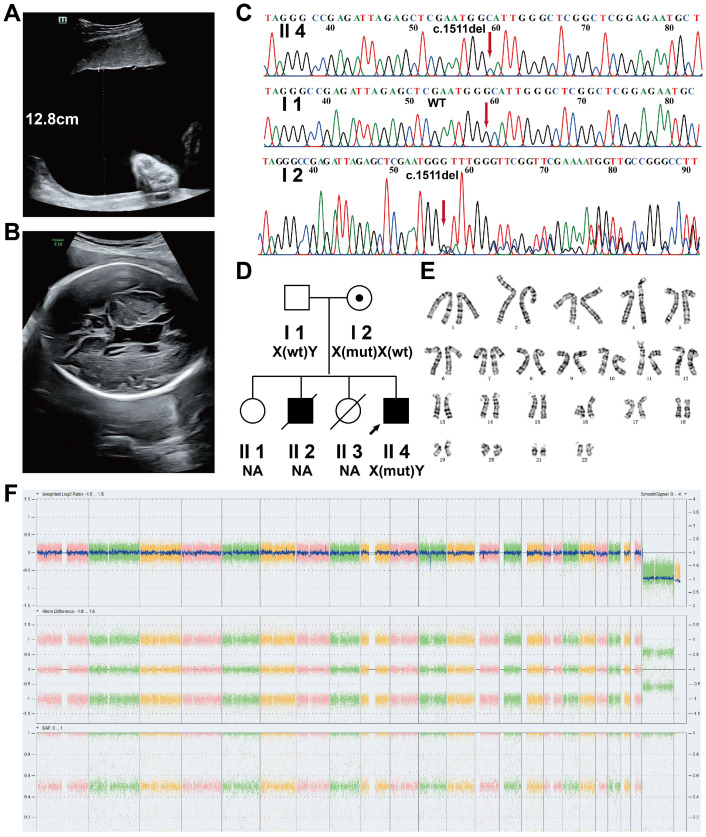
Clinical and genetic analyses of Case 1. (**A**) Prenatal ultrasound at 27 + 2 weeks of gestation showing polyhydramnios, with a maximum vertical pocket of 12.8 cm. (**B**) Ultrasound at 29 + 2 weeks demonstrating dilation of the left lateral ventricle (near field, 12.3 mm), right lateral ventricle (far field, 6.8 mm), and cavum vergae measuring approximately 10.6 mm, indicating enlargement of the left lateral ventricle and cavum vergae. (**C**) Sanger sequencing confirmation of the variant NM_177433.1 (*MAGED2*): c.1511del (p.Gly504Alafs*72). From top to bottom: hemizygous individual (II4), wild-type individual (I1), and heterozygous carrier (I2). (**D**) Pedigree of Case 1. (**E**,**F**) Chromosome analysis and CMA of the fetus (II4) revealed no abnormalities. The colors in panel F sequentially represent chromosomes (Chr1–Chr22, X, Y).

Case 1 involved a woman with four pregnancies. The pedigree of this family is shown in Figure 1D.

The first pregnancy resulted in the delivery of a healthy female infant without any reported complications (II1). The second pregnancy ended at 24 weeks of gestation due to severe polyhydramnios and placenta previa. A male infant was delivered by cesarean section but died shortly after birth (II2). The third pregnancy was terminated at 20 weeks of gestation, resulting in a stillborn female fetus with no obvious structural abnormalities (II3). The genotypes of individuals II1, II2, and II3 were unavailable.

The fourth pregnancy involved a male fetus (II4). Polyhydramnios was first detected at 20 + 3 weeks of gestation. Serial ultrasonographic examinations performed at 27, 28, 29, 30, 31, 32, and 34 weeks of gestation consistently demonstrated persistent polyhydramnios (Figure 1A). At 29 + 2 weeks, ultrasound revealed that the fetus was LGA, and the degree of fetal overgrowth progressively increased with advancing gestation. A total of four amnioreduction procedures were performed during pregnancy, with 1300–2500 mL of amniotic fluid removed at each procedure, resulting in a cumulative volume of 8300 mL.

Additional prenatal ultrasonographic findings included unilateral left lateral ventricular dilatation (first detected at 29 + 2 weeks, Figure 1B), enlargement of the cavum vergae (first detected at 29 + 2 weeks, Figure 1B), bilateral lateral ventricular dilatation (first detected at 31 + 1 weeks), enlargement of the cavum septi pellucidi (first detected at 31 + 1 weeks), bilateral scrotal enlargement (first detected at 31 + 1 weeks), increased fetal bowel echogenicity (first detected at 29 + 2 weeks), and fetal tachycardia (first detected at 27 + 2 weeks).

A cesarean section was performed at 33 weeks of gestation, resulting in the delivery of a live male neonate with an Apgar score of 10. Postnatal abnormalities included central atrial septal defect (detected at 4 months of age and resolved by 1 year and 8 months), lateral ventricular dilatation (detected at birth and improved by 1 year of age), bilateral hydrocele (detected at birth and resolved by 1 year), bilateral testicular enlargement (detected at 2 months and 22 days and resolved by 1 year, left testicular size 15 mm × 8 mm, right testicular size 15.6 mm × 10.5 mm), renal pelvic separation (detected at 2 months and 22 days and resolved by 1 year), communicating hydrocephalus (detected at 7 months), reduced corpus callosum volume (detected at 7 months), and formation of the fifth and sixth ventricles (detected at 7 days after birth). Follow-up until 3 years of age showed normal cognitive function and psychomotor development, with no additional abnormalities observed. Other clinical features within the family are summarized in Table S1.

Both chromosome analysis and CMA confirmed a male fetus with no detectable chromosomal abnormalities (Figure 1E,F) for the fourth fetus. ES, followed by Sanger sequencing, identified a maternally inherited c.1511del (p.Gly504Alafs*72) variant in the fetus (Figure 1C). This variant was classified as pathogenic (PVS1 + PM2_supporting + PP4).

#### 3.1.2. Case 2 [GRCh37] DEL: chrX:54,820,664–54,839,053 (Deletion Involving the 5′ End and Exons 1–7 of *MAGED2*)

**Figure 2:**
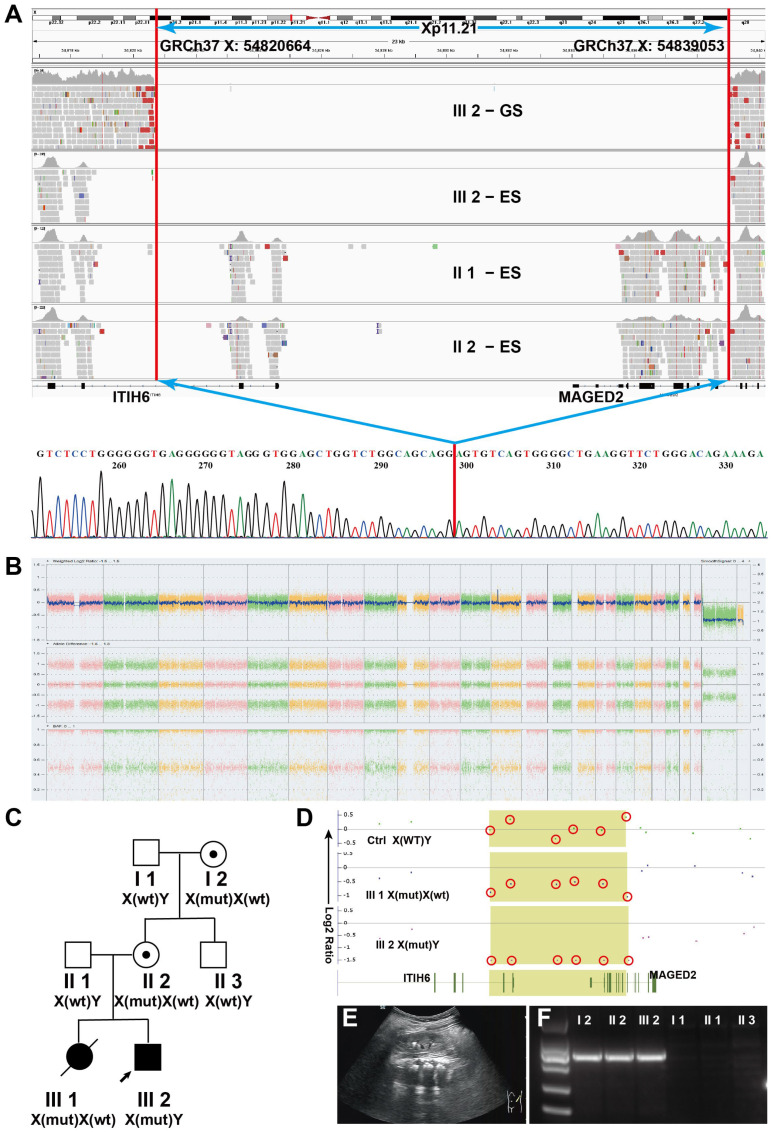
Clinical and genetic analyses of Case 2. (**A**) Identification of the variant [GRCh37] DEL: chrX-54,820,664–54,839,053. Read-depth analysis of GS and ES BAM files from individuals II1, II2, and III2 showed deletion signals involving *ITIH6* (Exon 1–Exon 2) and *MAGED2* (Exon 1–Exon 7) in individual II2 (upper panel). After determination of the breakpoint by Sanger sequencing, the deletion was confirmed as [GRCh37] DEL: chrX-54,820,664–54,839,053 (lower panel). (**B**) CMA performed prior to identification of the deletion showed no abnormalities. (**C**) Pedigree of Family 2. (**D**) After confirmation of the [GRCh37] DEL: chrX-54,820,664–54,839,053 variant in this family, reanalysis of CMA data from two fetuses (III1 and III2) revealed reduced signal intensity of six probes at the *MAGED2* and *ITIH6* loci, consistent with a microdeletion. (**E**) Renal calculus detected in the left kidney of individual III2 at 3 months of age. The colored boxes represent CMA data points in the deleted regions, and the red circles are used to highlight these data points. (**F**) Breakpoint junction analysis. A 703-bp fragment was amplified in individuals I2, II2, and III2, indicating carriage of the [GRCh37] DEL: chrX-54,820,664–54,839,053 variant, whereas no amplification was observed in individuals I1, II1, and II3, indicating absence of the deletion.

Case 2 involved a woman with two pregnancies. The pedigree is shown in Figure 2C.

At 23 gestational weeks, the first pregnancy (III1) was terminated due to polyhydramnios and premature rupture of membranes, resulting in the delivery of a female fetus. No additional abnormalities were detected during pregnancy. CMA initially revealed no abnormalities. After the delivery of the second fetus, a reanalysis of the CMA data was conducted, which revealed reduced signal intensity of six probes at the *MAGED2* locus, indicating that the first fetus also carried the [GRCh37] DEL: chrX:54,820,664–54,839,053 variant.

The second pregnancy (III2) involved a male fetus. Polyhydramnios was detected by ultrasound at 20 gestational weeks. Subsequent ultrasound examinations at 21, 25, 28, and 33 weeks consistently demonstrated polyhydramnios. LGA was identified from 20 weeks onward, with progressive fetal overgrowth as gestation advanced. A total of eight amnioreduction procedures were performed during pregnancy, with 1700–6700 mL of amniotic fluid removed each time, totaling 37,740 mL.

At 33 weeks of gestation, a healthy male infant was delivered by cesarean section with an Apgar score of 10. Postnatal abnormalities included atrial septal defect (detected at 3 months and resolved by 1 year), bilateral hydrocele (detected at birth and resolved by 3 months), hepatomegaly (detected at birth and resolved within 1 week), cardiomegaly (detected at birth and resolved within 1 week), and a left renal calculus detected at 3 months of age, which remained stable in size during follow-up to 4 years (Figure 2E). Neurodevelopmental outcomes were assessed during routine pediatric follow-up visits. At 4 years of age, the child demonstrated age-appropriate developmental milestones, as evaluated clinically by pediatricians according to standard age norms. A formal standardized developmental assessment tool was not administered. No additional abnormalities were observed. Other clinical features within the family are summarized in Table S1.

Both chromosome analysis and CMA showed no abnormalities for the second fetus (III2) (Figure 2B). Trio-ES identified a hemizygous deletion encompassing exons 1–7 of the *MAGED2* gene in the fetus, inherited from the heterozygous mother. GS followed by breakpoint junction analysis was conducted, which revealed a deletion spanning chrX:54,820,664–54,839,053. This deletion involved *ITIH6* (exons 1–2 and the 5′ untranslated region) and *MAGED2* (5′ untranslated region and exons 1–7), as shown in Figure 2A. The *MAGED2* deletion was classified as pathogenic (PVS1 + PM2_P + PP4). Currently, there are no reported human diseases associated with variants or deletions in *ITIH6*. In contrast, the clinical features observed in the fetus are consistent with those reported in association with *MAGED2* variants. Therefore, we consider the deletion involving *ITIH6* to be a clinically incidental finding rather than contributory to the phenotype.

Breakpoint junction analysis confirmed that the second male infant (III2), the mother (II2), and the maternal grandmother (I2) all carried the X-chromosomal deletion (Figure 2F). Subsequently, reanalysis of the CMA data from both fetuses (III1 and III2) demonstrated reduced signal intensity of six probes at the *MAGED2* and *ITIH6* loci, consistent with a microdeletion (Figure 2D III1 and III2), confirming that the first pregnancy was also affected.

#### 3.1.3. Case 3 c.338del (p.Pro113ArgfsTer4)

**Figure 3:**
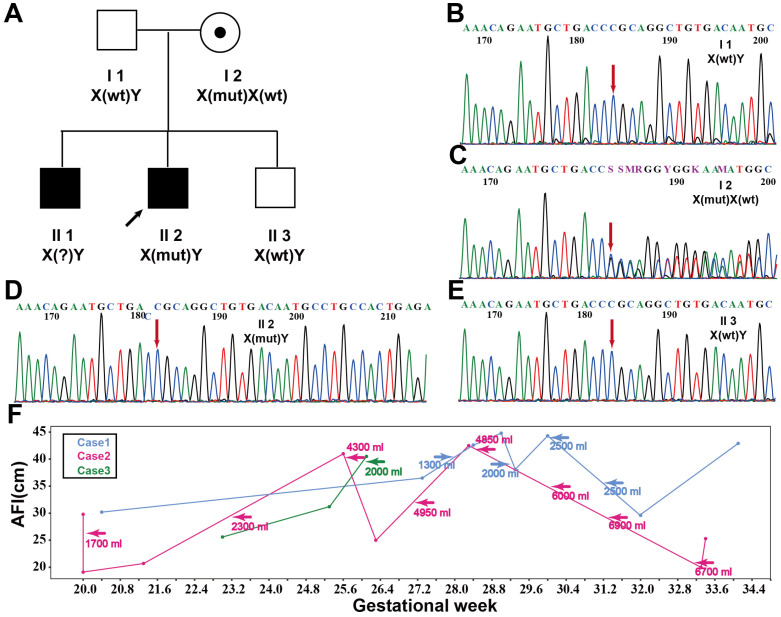
Clinical and genetic analyses of Case 3. (**A**) Pedigree of Family 3. (**B**–**E**) Sanger sequencing results showing that I1 and II3 are wild-type, I2 is heterozygous, and II2 is hemizygous. (**F**) Changes in AFI during pregnancy in the three families. Arrows indicate the time points at which amnioreduction was performed, with the volume of aspirated amniotic fluid recorded.

Case 3 involved a woman with three pregnancies. The pedigree is shown in Figure 3A.

At 24 gestational weeks, spontaneous abortion occurred due to polyhydramnios, resulting in the delivery of a male stillbirth. No genetic testing was performed for the first fetus (II1).

The second fetus (II2) was male. Polyhydramnios was detected at 23 + 0 weeks. Magnesium sulfate and ritodrine were administered during pregnancy. At 26 + 1 weeks, an amnioreduction procedure was performed, with 2000 mL of amniotic fluid removed. Premature rupture of membranes occurred at 26 + 1 weeks, resulting in the delivery of a male stillbirth. CMA revealed no abnormalities. Retrospective analysis during the third pregnancy demonstrated that this fetus carried a hemizygous *MAGED2*:c.338del (p.Pro113ArgfsTer4) variant (Figure 3D).

ES was first performed on the pregnant woman and her husband, revealing that the mother carried a heterozygous *MAGED2*:c.338del (p.Pro113ArgfsTer4) variant, which was classified as pathogenic according to ACMG guidelines (PVS1 + PM2_supporting + PP4). Given the history of two prior pregnancies complicated by polyhydramnios and spontaneous abortion, Sanger sequencing was performed in the second aborted fetus (II2), the third (current) fetus (II3), the mother, and the father (Figure 3B–E). The c.338del variant was detected in the second aborted fetus (Figure 3D). CMA and chromosome analysis of the current fetus revealed a karyotype of 46,XY,inv(9)(p12q13), which is considered a benign polymorphic variant, with no additional chromosomal abnormalities detected. No polyhydramnios occurred during this pregnancy, and a healthy male infant was delivered.

#### 3.1.4. Pregnancy-Related Amniotic Fluid Changes and Amnioreduction: 3 Case Reports

Since polyhydramnios is a major symptom of TABS, we analyzed the changes in amniotic fluid volume during pregnancy in three cases. Additionally, we examined the timing of amnioreduction procedures and the volume of amniotic fluid removed. Detailed measurements of amniotic fluid volume and the amount removed at each procedure are shown in Figure 3F. 

### 3.2. Null Variants Are the Predominant Pathogenic Variant Type in MAGED2

Including the present study, a total of 53 affected individuals have been reported. We summarized the types of *MAGED2* variants, the sex of affected individuals, prenatal manifestations, prenatal treatments, pregnancy outcomes, and postnatal phenotypes of previously reported families. (Table S2). To date, 38 distinct *MAGED2* variants have been described, including 10 frame-shift, 7 nonsense, 6 splice-site, 6 missense, 4 in-frame indels, 2 exon deletions, and 1 whole-gene deletion (Table S3).

**Figure 4:**
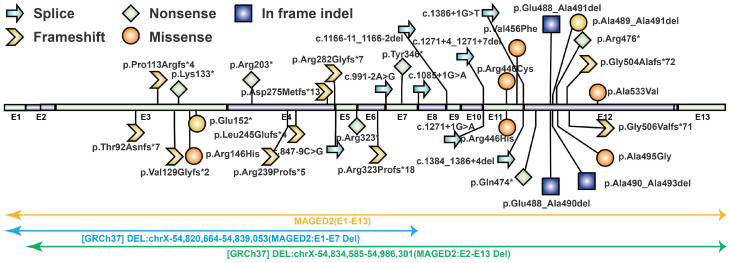
Previously reported pathogenic *MAGED2* variants. A total of 38 pathogenic *MAGED2* variants have been reported to date, including 10 frameshift variants, 4 in-frame indels, 6 missense variants, 7 nonsense variants, 8 splice-site variants, 2 exon deletions, and 1 whole-gene deletion. Exon 12 harbors the highest number of variants (*n* = 10). * indicates a stop codon.

Null variants accounted for 65.8% (25/38) of all reported variants and represented the predominant pathogenic variant type. Variants were mainly distributed in exons 11 and 12 (Figure 4). Regarding inheritance, most variants were maternally inherited (70%, 39/53), while de novo variants accounted for 12.5% (5/53), and one case was inherited from the father (1.9%, 1/53) (Table S2).

### 3.3. Polyhydramnios Is the Major Prenatal Manifestation

Polyhydramnios was observed in all 53 reported patients, most commonly detected at 19–20 gestational weeks (37.7%, 20/53). LGA was the second most frequent prenatal finding (34.0%, 18/53). Other rare prenatal ultrasound findings (≤2 reported cases each) included cavum vergae enlargement, bilateral ventriculomegaly, enlarged cavum septi pellucidi, increased bilateral scrotal volume, fetal tachycardia, suspected urethral anomalies, hyperechogenic foci in the left ventricle and bowel, vascular developmental abnormalities, tetralogy of Fallot, microretrognathia with low-set ears, cleft palate, and esophageal atresia.

### 3.4. Amnioreduction Is the Main Therapeutic Intervention

Because clinical management and delivery information were relatively complete for cases 1–25 and 27–28 in Table S2, these 27 cases were included in further analysis. Among them, 18 pregnant women underwent amnioreduction, of whom 14 delivered live infants (77.8%, 14/18). Among the nine women who did not undergo amnioreduction, seven delivered live infants (77.8%, 7/9). In addition to amnioreduction, other treatments included indomethacin, betamethasone, ritodrine hydrochloride, magnesium sulfate, dexamethasone, and atosiban.

### 3.5. Transient Polyuria Is the Most Common Postnatal Manifestation

A total of 41 surviving infants were identified from Table S2. Among them, 30 developed transient postnatal polyuria (73.1%, 30/41), 27 exhibited transient electrolyte abnormalities (65.9%, 27/41), and 8 developed nephrocalcinosis (19.5%, 8/41). Other reported manifestations (each in fewer than three cases) included elevated plasma renin and aldosterone levels, hypotonia, septal defects, ventriculomegaly, bilateral hydrocele, bilateral testicular enlargement, renal pelvis separation, communicating hydrocephalus, reduced corpus callosum volume, variants of the cavum septi pellucidi and cavum vergae, hepatomegaly, cardiomegaly, motor delay, mild peripheral hypertonia, angiomas, and Fanconi renotubular syndrome. Notably, no cases with intellectual disability have been reported to date.

## 4. Discussion

In addition to the present study, 13 previously published articles have reported a total of 53 cases of TABS. Severe polyhydramnios represents the most consistent prenatal manifestation of TABS. LGA has been reported as the second most common feature [12,14], which was also observed in the cases described in our cohort. However, polyhydramnios may also occur as an isolated prenatal finding in TABS [1,11,15,17,18,19,20,21,22]. Buffet et al. reported that oesophageal atresia, tetralogy of Fallot, microretrognathia, low-set ears, and suspected cleft palate may occur in TABS [11]. In addition, other prenatal abnormalities described in the literature include urethral anomalies [14], hyperechogenic foci in the left ventricle and fetal bowel [13], and vascular anomalies [23]. In our study, additional findings were observed, including left lateral ventricular dilatation, enlarged cavum vergae, bilateral lateral ventricular dilatation, enlarged cavum septi pellucidi, increased bilateral scrotal volume, increased fetal intestinal echogenicity, and fetal tachycardia. Although these additional findings cannot be definitively attributed to *MAGED2* variants, they warrant careful attention during prenatal assessment and postnatal follow-up.

A comprehensive review of the existing literature reveals that premature rupture of membranes (PROM), which often results in preterm birth, represents a prevalent obstetric complication in affected pregnancies. Furthermore, polyuria and electrolyte disturbances are frequently observed as postnatal clinical manifestations of TABS. Buffet et al. followed three cases (1 year, 1.5 years, and several years) [11]. Walsh et al. followed one case until 7 months [14]. Xu et al. followed one case until 20 months [21]. Yang et al. followed one case until 12 months [13]. Ma et al. followed one case until 4 years [18]. Arthuis et al. followed one case until 12 months [23]. Legrand et al. followed 13 cases for 0.4–12.5 years [12]. Laghmani et al. reported follow-up of one case until 17 years of age [1]. The available literature consistently indicates an absence of reported intellectual developmental abnormalities, suggesting that TABS does not exhibit significant adverse effects on cognitive development. In this study, two surviving cases of TABS were monitored for follow-up periods of 4 years and 1.5 years, respectively, with no evidence of intellectual disability observed in either case. These findings align with prior research, which has consistently reported normal neurodevelopmental outcomes in long-term follow-up assessments.

In addition to TABS, Beckwith–Wiedemann syndrome (BWS), Simpson–Golabi–Behmel syndrome (SGBS), and unrecognized gestational diabetes mellitus (GDM) may also present with polyhydramnios accompanied by fetal macrosomia or LGA. TABS is typically characterized by early-onset and severe polyhydramnios, and male fetuses are at increased risk of being affected. These features provide important diagnostic clues during prenatal evaluation [11]. Although all four conditions can result in polyhydramnios and fetal overgrowth, BWS and SGBS are usually associated with distinctive facial features, abnormal body habitus, or visceral malformations [24,25]. In contrast, GDM commonly manifests as disproportionate truncal overgrowth and increased abdominal circumference. Moreover, maternal hyperglycemia is a key distinguishing factor for the diagnosis of GDM [26]. Notably, a positive family history may be observed in cases of TABS and SGBS, which may further assist in the differential diagnosis [27].

Amnioreduction can alleviate maternal symptoms and is considered a relatively safe procedure with a low rate of complications [28]. Comparison of live-birth rates between pregnancies managed with amnioreduction and those receiving other treatments revealed no significant difference, suggesting that the efficacy of these interventions in aBS requires further evaluation. Walsh et al. reported that amnioreduction was performed at 23, 26, and 29 weeks of gestation, during which 1900 mL, 1400 mL, and 1900 mL of amniotic fluid were removed, respectively, and a male infant was delivered at 37 + 6 weeks of gestation [14]. These findings were compared with the gestational age–dependent AFI measurements and the volumes of amnioreduction plotted in the present study. Aside from the timing of intervention, no clear differences were observed between live-birth and stillbirth cases (Figure 3F). Importantly, in Cases 1 and 2, which resulted in live births, amnioreduction was initiated earlier than in Case 3, which ended in fetal demise. Moreover, the diagnosis of *MAGED2*-related polyhydramnios was established earlier in Cases 1 and 2 than in Case 3. Although early recognition and intervention may facilitate closer monitoring, the current data do not allow definitive conclusions regarding the impact of amnioreduction on fetal survival.

We further summarized the spectrum of pathogenic *MAGED2* variants and identified three cases involving exon deletions, a variant type that may be missed if exon-level coverage analysis and CNV-aware pipelines are not systematically evaluated [17,29]. In Case 2, the exon deletion was also present in the first pregnancy but remained undetected during the initial evaluation and was only identified retrospectively after the second affected pregnancy. In this case, careful examination of exon-level read depth in ES BAM files, probe signal intensity in CMA, and confirmatory analyses using GS and Sanger sequencing were required to establish the diagnosis. These findings highlight the importance of scrutinizing exon-level coverage and copy-number signals in fetuses with severe polyhydramnios—particularly when the AFI is markedly elevated—to avoid missed diagnoses of *MAGED2* exon deletions.

Pathogenic *MAGED2* variants are predominantly maternally inherited, and affected individuals are overwhelmingly male (95%, 38/40; Table S2). Nevertheless, two female fetuses carrying pathogenic variants and presenting with polyhydramnios have been reported [22,29]. In the present study, we also identified a female fetus harboring a pathogenic *MAGED2* variant who developed polyhydramnios and did not survive (Figure 2D, III1). Although rare, female fetuses with pathogenic variants have been reported; therefore, TABS may be considered in selected cases of unexplained severe polyhydramnios regardless of fetal sex.

Although this study summarized the clinical data of 53 reported TABS cases up to the end of 2025, the sample size remains limited. In addition, incomplete clinical information in some reports further exacerbates this limitation. The small sample size restricts our ability to fully evaluate the effectiveness of prenatal interventions and to assess the frequency of certain rare clinical features. Furthermore, although the pathogenicity of the identified *MAGED2* variants was evaluated according to ACMG guidelines, functional studies were not performed. Larger TABS cohorts and functional investigations are essential to clarify the pathogenic mechanisms and clinical characteristics of the disease. Such efforts are critically important for improving the diagnosis of TABS, guiding prenatal management, and informing postnatal care.

## 5. Conclusions

In conclusion, we report three previously unreported pathogenic variants in TABS, thereby expanding the known mutational spectrum of this gene. Based on a summary of previously reported cases together with our patients, polyhydramnios appears to be the most prominent prenatal manifestation, while large for gestational age (LGA) represents the second most frequently observed feature.

Among the reported cases, amnioreduction has been the most commonly applied therapeutic intervention for managing severe polyhydramnios. However, due to the limited sample size, the relative effectiveness of amnioreduction compared with other prenatal interventions cannot yet be reliably evaluated. Whether early detection and timely amnioreduction can improve fetal survival requires confirmation in larger cohorts.

Furthermore, in fetuses presenting with early-onset severe polyhydramnios (around 19–20 weeks of gestation), particular attention should be paid to possible exon-level or partial deletions involving *MAGED2* during genetic evaluation.

## Data Availability

The original contributions presented in this study are included in the article/Supplementary Materials. Further inquiries can be directed to the corresponding authors. There was no code used for this study.

## Supplementary Materials

The following supporting information can be downloaded at: https://www.mdpi.com/article/10.3390/genes17040424/s1, Table S1: Clinical characteristics of the three cases; Table S2 Case reports of individuals with aBS; Table S3: Reported *MAGED2* variants to date.

## Abbreviations

The following abbreviations are used in this manuscript:

TABS	Transient antenatal Bartter syndrome
TAL	Thick ascending limb
CMA	Chromosome microarray analysis
ES	Exome sequencing
GS	Genome sequencing
LGA	Large for gestational age
