# Supplementary material for: Identification of Three Novel MAGED2 Variants Causing Antenatal Bartter Syndrome in Three Chinese Families
Source: Genes (Basel). 2026 Apr 3;17(4):424. doi: 10.3390/genes17040424 (PMC13116143; doi:10.3390/genes17040424)
Supplement: Supplementary file 1 [file genes-17-00424-s001.zip › genes-4195316-supplementary.pdf]

Table S1. Clinical characteristics of the three cases

	Case 1	Case 2	Case 3
**Fetal gender**	Male	Male	Male
**Variants**	c.1511del(p.Gly504Alafs*72)	[GRCh37] DEL:chrX-54,820,664-54,839,053	c.338del(p.Pro113ArgfsTer4)
**History of gestation**	Four pregnancies in total. The first resulted in the delivery of a healthy female infant with an uneventful perinatal course (genotype unknown). The second pregnancy was terminated at 20 gestational weeks, resulting in a female stillbirth with no apparent anomalies (genotype unknown). The third pregnancy resulted in the delivery of a male infant by caesarean section at 24 gestational weeks due to polyhydramnios and placenta previa; the neonate died shortly after birth (genotype unknown). The fourth pregnancy, corresponding to the fetus described in the present study, was also complicated by polyhydramnios and other obstetric complications; this fetus was found to carry a pathogenic MAGED2 variant.	Two pregnancies in total. The first pregnancy ended at 23 gestational weeks with the miscarriage of a female infant due to polyhydramnios and preterm premature rupture of membranes; the fetus carried a pathogenic MAGED2 variant. The second pregnancy, which is described in detail in the present study, also involved a fetus carrying a pathogenic MAGED2 variant and was complicated by polyhydramnios and other obstetric complications.	Three pregnancies in total. The first two involved male fetuses and both pregnancies ended in miscarriage due to polyhydramnios. The second miscarriage is described in detail in the present study; the fetus carried a pathogenic MAGED2 variant, whereas the genotype of the first miscarried fetus was unknown. The third pregnancy involved a male fetus without a pathogenic MAGED2 variant and proceeded uneventfully, resulting in the delivery of a healthy male infant.
**Gestational manifestations**			

**Polyhydramnios (amniotic fluid index, cm)**	20w+3 (30.2)<br>27w+2 (36.5)<br>28w+3 (42.6)<br>29w+0 (44.8)<br>29w+2 (38.1)<br>30w+0 (44.3)<br>31w+0 (37.0)<br>32w+0 (29.6)<br>34w+1 (42.9)	20w+0 (29.8)<br>20w+1 (19.1)<br>21w+2 (20.7)<br>25w+4 (41.0)<br>26w+2 (25.0)<br>28w+2 (42.5)<br>33w+2 (20.0)<br>33w+3 (25.3)	23w+0(25.6)<br>25w+2(31.2)<br>26w+1(40.5)
**Large for gestational age***	29w+2: 32w+d (HC), 31w+5 (FL)<br>32w+0: 36w+2 (HC), 33w+0 (FL), 35w+0 (AUA)<br>34w+1: 40w+0 (HC), 36w+5 (FL), 39w+5 (AUA)	20w+0: 20w+2 (HC), 21w+3 (AC), 21w+1 (FL), 20w+6 (AUA)<br>21w+2: 21w+3 (HC), 21w+6 (FL), 21w+4 (AUA)<br>25w+4: 26w+6 (HC), 27w+6 (AC), 26w+5 (FL), 27w+1 (AUA)<br>26w+2: 27w+5 (HC), 27w+1 (FL), 27w+3 (AUA)<br>28w+2: 30w+5 (AUA)<br>33w+3: 37w+4 (HC), 38w+5 (AC), 35w+2 (FL), 37w+1 (AUA)<br>33w+2: 36w+5 (HC), 35w+0 (FL), 36w+4 (AUA)	NA
**Left lateral ventricular dilatation**	Detected by ultrasonography at 29w+2, 32w, 34w+1	None	NA
**Bilateral lateral ventricular dilatation**	Detected by MRI at 31w+1	None	NA

**Enlarged cavum septi pellucidi**	Detected by MRI at 31w+1	None	NA
**Enlarged cavum vergae**	Detected by ultrasonography at 29w+2, 32w, 34W+1	None	NA
**Bilateral scrotal enlargement**	Detected by MRI at 31w+1	None	NA
**Fetal bowel echogenicity**	Detected by ultrasonography at 29w+2, 32w	None	NA
**Fetal tachycardia**	Detected by ultrasonography at 27w+2	None	NA
**Amnioreduction(ml)**	28w+1 (1300)<br>29w+1 (2000)<br>30w+1 (2500)<br>31w+1 (2500)<br>A total of four surgeries.	20w+1 (1700)<br>23w+2 (2340)<br>25w+4 (4300)<br>27w+0 (4950)<br>28w+3 (4850)<br>30w+0 (6000ml)<br>31w+3 (6900ml)<br>33w+0 (6700ml)<br>A total of eight surgeries.	26w+1(2000)<br>A single surgery in total.
**Pregnancy outcome**	Cesarean delivery at 33 weeks of gestation; Apgar score 10; no other abnormalities observed.	Cesarean delivery at 33 weeks of gestation; Apgar score 10	Preterm premature rupture of membranes at 26 + 2 weeks, resulting in inevitable miscarriage; male fetus with no apparent external anomalies.
**Postnatal follow-up**			
**Central atrial septal defect**	Detected at 4 months of age and resolved by 1 year and 8 months	Atrial septal defect (small) detected at 3 months of age and resolved by 1 year	NA

**(ostium secundum type)**			
**Lateral ventricular dilatation**	Detected at birth and at 1 year of age; milder at 1 year compared to birth	None	NA
**Bilateral testicular hydrocele**	Detected at birth and resolved by 1 year of age	Detected at birth and resolved by 3 months of age	NA
**Bilateral testicular enlargement**	Detected at 2 months and 22 days of age; resolved by 1 year	None	NA
**Renal sinus separation**	Detected at 2 months and 22 days of age; resolved by 1 year	None	NA
**Electroencephalography**	Normal	Normal	NA
**Communicating hydrocephalus**	Detected by MRI at 7 months of age	None	NA
**Reduced corpus callosum volume**	Detected by MRI at 7 months of age	None	NA
**Formation of the fifth and sixth ventricles**	Detected by MRI at 7 days of age	None	NA
**Hepatomegaly**	None	Detected at birth and resolved within 1 week	NA
**Cardiomegaly**	None	Detected at birth and resolved within 1 week	NA
**Renal calculus**	None	Appeared at 3 months of age; followed up until 4 years, with no increase in calculi reported	NA

Genetic testing			
**CNV**	Negative	Negative	Negative
**Karyotype**	Negative	Negative	Negative
**WES**	NM_177433.3: c.1511del(p.Gly504Alafs*72), Hemizygous	chrX:54820665-54839050 (Exon 1-7 of MAGED2 del), Hemizygous	NM_177433.1: c.338del(p.Pro113Argfs*4), Hemizygous
**ACMG**	PVS1+PM2_supporting+PP4 (Pathogenic)	PVS1+PM2_supporting+PP4 (Pathogenic)	PVS1+PM2_supporting+PP4 (Pathogenic)
**Inherited**	mat	mat	mat

***Fetal gestational age estimated at different gestational weeks based on HC, FL, AUA, and AC**

Table S2 Case reports of individuals with aBS

Case	Variants and inheritance	Gender	Perinatal manifestations	Treatment	Pregnancy outcome	Postnatal follow-up
1#	c.1511del(p.Gly504Alafs*72), mat	Male	Polyhydramnios (20 + 3 w), fetal macrosomia, left lateral ventricular dilatation, enlarged cavum vergae, bilateral lateral ventricular dilatation, enlarged cavum septi pellucidi, increased bilateral scrotal volume, increased fetal intestinal echogenicity, and fetal tachycardia	Amnioreduction	a male infant, cesarean, 33 w	Central atrial septal defect, ventricular enlargement, bilateral testicular hydrocele, bilateral testicular enlargement, renal sinus separation, communicating hydrocephalus, reduced corpus callosum volume, and formation of the fifth and sixth ventricles. Followed up until 3 years of age, with normal intelligence and development and no other abnormalities observed.

2#	[GRCh37] DEL:chrX-54,820,664-54,839,053(MAGED2:E1-E7 Del), mat	Male	Polyhydramnios (20 w) and Large for gestational age	Amnioreduction	a male infant, cesarean, 33 w	Polyuria (first 3 days after birth), atrial septal defect, hepatomegaly, cardiomegaly, and kidney stones. Followed up until 4 years of age, with normal intelligence and development and no other abnormalities observed.
3#	c.338del(p.Pro113Argfs*4), mat	Male	Polyhydramnios (23 w) and Large for gestational age (20 w)	Amnioreduction	a male stillbirth, premature rupture, 26+1 w	NA
4[1]	c.1336C>T(p.Arg446Cys),mat	Male	Polyhydramnios (24.5 w)	NA	a male infant, 26 w	Mild motor delay and light peripheral hypertonia(Followed up until 1.5 years of age)
5[1]	c.1466-1474del(p.Ala489_Ala491del),mat	Male	Polyhydramnios	NA	a male infant, 26 w	NA
6[1]	c.1166-11_1166-2del,mat	Female	Polyhydramnios (22 w)	Amnioreduction	a female infant, 24 w	Postnatal death
7[1]	c.1166-11_1166-2del,mat	Male	Polyhydramnios (25.3w)	Amnioreduction	a male infant, 25.3 w	Postnatal death
8[1]	c.1426C>T(p.Arg476*),mat	Male	Polyhydramnios (24w)	NA	a male infant, 27w	Angiomas (back,face and severe hepatic hemangiomatosis)(Followed up until 1 years of age)
9[1]	c.1337G>A(p.Arg146His),?	Male	Polyhydramnios	Amnioreduction	a male infant, 34w	No apparent abnormalities

10-11[1]	c.454G>T(p.Glu152*),mat	Male	Polyhydramnios (19w)	Amnioreduction	a male infant, 19w	Postnatal death
12[1]	c.454G>T(p.Glu152*),mat	Male	Polyhydramnios (19w)	Amnioreduction	a male infant, 30w	No apparent abnormalities
13[1]	c.1464_1475del(p.Ala490_Ala493del),mat	Male	Polyhydramnios (24w), Oesophageal Atresia	Amnioreduction	intrauterine fetal demise, 26w	NA
14[1]	c.1464_1475del(p.Ala490_Ala493del),mat	Male	Polyhydramnios (18w), Fallot tetralogy discovered at 18SA, microretrognathia, low-set ears, suspected cleft palate	Amnioreduction	induced abortion, 25w	NA
15[1]	c.716_732del(p.Arg239Profs*5),pat	Male	Polyhydramnios (22w)	Amnioreduction	a male infant, 36w	No apparent abnormalities
16[1]	c.1386+1G>T, mat	Female	Polyhydramnios (24w)	None	a female infant, 36w	Fanconi renotubular syndrome(Followed up until 5 years of age)
17[2]	c.1271+4_1271+7delAGTA, *de novo*	Male	Polyhydramnios (20w), premature rupture of membranes (25 w)	Magnesium sulfate	a male infant, 32w, survived for 8 hours	NA
18[2]	c.1271 + 4_1271 + 7delAGTA, *de novo*	Female	Polyhydramnios (20w), premature rupture of membranes (25 w)	Indomethacin, Amnioreduction, atosiban。	a female infant, 38w	No apparent abnormalities
19[3]	c.1337G>A, p.Arg446His, mat	Male	Polyhydramnios (20w), Large for gestational age, Suspected urethral abnormality.	None	a male stillbirth, 25w	NA

20[3]	c.1337G>A, p.Arg446His,mat	Male	Polyhydramnios (22w), Large for gestational age	Amnioreduction	a male infant, 37w+6	No apparent abnormalities (Followed up until 7 months of age)
21[4]	c.967C>T(p.Asp323*),mat	Male	Polyhydramnios (25w)	Magnesium sulfate, Indomethacin, dexamethasone, Amnioreduction	a male infant, 34w+6	No apparent abnormalities (Followed up until 20 months of age)
22[5]	c.1426C>T(p.Arg476*),mat	Male	Polyhydramnios (24w), Echogenic foci in the left ventricle and intestines.	Dexamethasone	a male infant, 27w+3	Polyuria (first 3 days after birth) with transient low levels of sodium, chloride, and potassium, which later normalized; followed up until 12 months with no abnormalities observed
23[6]	c.733_734delCT(p.Leu245Glufs*4),mat	Male	Polyhydramnios (21w)	Amnioreduction	a male infant, 35w+2	No apparent abnormalities (Followed up until 2 months of age)
24[7]	c.1085+1G>A,mat	Male	Polyhydramnios (19w)	Betamethasone, Ritodrine hydrochloride, Magnesium sulfate, Amnioreduction	a male infant, cesarean, 31w+6	Polyuria after birth, which later normalized; no other abnormalities observed; no follow-up available
25[8]	c.1598C>T(p.Ala533Val), mat	Male	Polyhydramnios (22w)	None	a male infant, 29w	Polyuria, dehydration, hyponatremia, hypokalemia, hypochloremia, and metabolic alkalosis after birth, all resolved within 2 months; followed up until

						4 years of age with no other abnormalities observed
26[9]	[GRCh37] DEL:chrX-54,834,585-54,986,301(MAGED2:E2-E13 Del), mat	Male	Polyhydramnios (20w)	NA	a male infant, 25w	No apparent abnormalities
27[10]	c.823delG(p.Asp275Metfs*13), mat	Male	Polyhydramnios (19w), a right aortic arch with a slight isthmic stenosis, a moderate pulmonary stenosis and a left subclavian artery arising from a Kommerel diverticulum, large for gestational age.	Amnioreduction	a male infant, 36w	Postnatal hyperhydration, resolved after 4 days; moderate sitting hypotonia and peripheral hypertonia of the lower limbs observed at 9 months; followed up until 12 months with no other abnormalities observed
28[11]	MAGED2##	Male	Polyhydramnios (21w)	Indomethacin, Amnioreduction	a male infant, 29w	Polyuria, hypokalemia, and hyponatremia after birth, normalized after 29 days; increased renal echogenicity, improving from day 14; elevated plasma renin and aldosterone; followed up until 29 days with no other abnormalities observed

29-44[12]	c.607C>T(p.Arg203*),*de novo*<br>c.842_843dup(p.Arg282Glyfs*7), mat<br>c.967C>T(p.Arg323*), mat<br>c.967dup(p.Arg323Profs*18), ?<br>c.1085+1G>A, *de novo*<br>c.1271+1G>A, ?<br>c.1336C>T(p.Arg446Cys), mat<br>c.1337G>A(p.Arg446His), mat<br>c.1366G>T(p.Val456Phe), mat<br>c.1384_1386+4del, mat<br>c.1420C>T(p.Gln474*), mat<br>c.1420C>T(p.Gln474*), mat（female）<br>c.1458_1466del(p.Glu488_Ala490del), ?<br>c.1464_1475del(p.Ala490_Ala493del), ?<br>c.1515_1516dup(p.Gly506Valfs*71), mat<br>MAGED2(E1-E13), mat	15 Male<br>1 Female	All 16 pregnant women presented with polyhydramnios (22–25 weeks). 12 pregnant women presented with polyhydramnios.	Amnioreduction was performed in 11 pregnant women. Indomethacin was administered to 4 pregnant women.	Sixteen pregnant women delivered preterm (26–33 w), including one by cesarean section	All neonates presented with polyuria after birth. Follow-up was performed for 13 neonates (0.4–12.5 years), with water and electrolyte loss resolving between 2 and 18 months. Two neonates died: the fetus with c.842_843dup(p.Arg282GlyfsTer7) died at 1 month, accompanied by thrombocytopenia; the fetus with c.1271+1G>A died at 12 months, presenting with leukomalacia, severe dehydration, and hypernatremia.
45[13]	c.1038C>G(p.Tyr346*), mat	Male	Polyhydramnios (19w)	NA	deliveries at 22w, 27w, and 31 w	Fetus delivered at 22 weeks, postnatal death. Fetuses delivered at 27 and 32 weeks presented with polyuria, resolving at 5 weeks and 1 week, respectively. Renal calcifications observed, accompanied by salt-wasting symptoms that later resolved. The 27-week neonate received indomethacin within the first year

						after birth. Followed up until 17 years of age with no other abnormalities observed.
46[13]	c.1462_1473del(p.Glu488_Ala491del), mat	Male	Polyhydramnios (20w)	NA	deliveries at 25w and 28w	Fetus delivered at 25 weeks, postnatal death. The 28-week fetus presented with polyuria lasting until 5 weeks. Apart from abnormal blood electrolyte levels, no other abnormalities were reported.
47[13]	c.991-2A>G, mat	Male	Polyhydramnios (19w)	NA	deliveries at 34w, 33w, and 31w	All three fetuses survived. Polyuria was reported in the 31- and 33-week fetuses, both treated with indomethacin. Apart from abnormal blood electrolyte levels, no other abnormalities were reported.
48[13]	c.1484C>G(p.Ala495Gly), mat	Male	Polyhydramnios (20w)	NA	a male infant, 24w	Polyuria persisted for six weeks. Renal calcifications observed. Treated with indomethacin. Apart from abnormal blood electrolyte levels, no other abnormalities were reported.

49[13]	c.274dupA(p.Thr92Asnfs*7), ?	Male	Polyhydramnios (20w)	NA	a male infant, 26w	Polyuria persisted for 4 weeks. Renal calcifications observed. Apart from abnormal blood electrolyte levels, no other abnormalities were reported.
50[13]	c.397A>T(p.Lys133*),*de novo*	Male	Polyhydramnios (19w)	NA	a male infant, 27w	Polyuria persisted for 4 weeks. Renal calcifications observed. Treated with indomethacin. Apart from abnormal blood electrolyte levels, no other abnormalities were reported.
51[13]	c.386_87delTG(p.Val129Glyfs*2), mat	Male	Polyhydramnios	NA	deliveries at 29w and 30w	Fetus delivered at 30 weeks, postnatal death. The 29-week fetus presented with polyuria lasting until 1 week, with renal calcifications observed. Treated with indomethacin. Apart from abnormal blood electrolyte levels, no other abnormalities were reported.
52[13]	c.1336 C>T(p.Arg446Cys), mat	Male	Polyhydramnios (19w)	NA	a male infant, 22w	Postnatal death
53[13]	c.847-9C>G, mat	Male	Polyhydramnios (19w)	NA	a male infant, 29w	No apparent abnormalities

This report

The study only reported aBS caused by MAGED2 variants without specifying the exact variant

Reference

[1] Buffet A, Filser M, Bruel A, et al. X-linked transient antenatal Bartter syndrome related to MAGED2 gene: Enriching the phenotypic description and pathophysiologic investigation[J]. Genet Med, 2025, 27(2): 101217.

[2] Yan X, Hu Y, Zhang X, et al. Identification of a novel intronic mutation of MAGED2 gene in a Chinese family with antenatal Bartter syndrome[J]. BMC Med Genomics, 2024, 17(1): 23.

[3] Walsh CJ, Micke K, Elfman H, et al. Successful antenatal treatment of MAGED2-related Bartter syndrome and review of treatment options and efficacy[J]. Prenat Diagn, 2024, 44(2): 172-179.

[4] Xu K, Zhang YQ, Hou XL, et al. A case with prenatal molecular diagnosis of X-linked transient antenatal Bartter syndrome[J]. Journal of Translational Genetics and Genomics, 2023, 7(2): 87-93.

[5] Yang H, Liu Z, Wu Y, et al. Case Report: Transient antenatal bartter syndrome in an extremely preterm infant with a novel MAGED2 variant[J]. Front Pediatr, 2022, 10: 1093268.

[6] Wu X, Huang L, Luo C, et al. A Case Report and Literature Review of a Novel Mutation in the MAGED2 Gene of a Patient With Severe Transient Polyhydramnios[J]. Front Pediatr, 2021, 9: 778814.

[7] Takemori S, Tanigaki S, Nozu K, et al. Prenatal diagnosis of MAGED2 gene mutation causing transient antenatal Bartter syndrome[J]. Eur J Med Genet, 2021, 64(10): 104308.

[8] Ma M, Zhang M, Zhou Y, et al. A novel MAGED2 variant in a Chinese preterm newborn with transient antenatal Bartter's syndrome with 4 years follow-up[J]. BMC Nephrol, 2021, 22(1): 408.

[9] Yang K, Huo X, Zhang Y, et al. [Genetic analysis of a pedigree affected with Bartter's syndrome][J]. Zhonghua Yi Xue Yi Chuan Xue Za Zhi, 2019, 36(7): 701-703.

[10] Arthuis CJ, Nizon M, Komhoff M, et al. A step towards precision medicine in management of severe transient polyhydramnios: MAGED2 variant[J]. J Obstet Gynaecol, 2019, 39(3): 395-397.

[11] Meyer M, Berrios M, Lo C. Transient Antenatal Bartter's Syndrome: A Case Report[J]. Front Pediatr, 2018, 6: 51.

[12] A. Legrand, C. Treard, I. Roncelin, et al., Prevalence of Novel MAGED2 Mutations in Antenatal Bartter Syndrome. Clin J Am Soc Nephrol, 2018. 13(2): p. 242-250.

[13] Laghmani K, Beck BB, Yang SS, et al. Polyhydramnios, Transient Antenatal Bartter's Syndrome, and MAGED2 Mutations[J]. N Engl J Med, 2016, 374(19): 1853-1863.

Table S3 Reported MAGED2 variants to date

ID	Variant	Type
1	p.Thr92Asnfs*7	Frameshift
2	p.Pro113Argfs*4	Frameshift
3	p.Val129Glyfs*2	Frameshift
4	p.Lys133*	Nonsense
5	p.Arg146His	Missense

6	p.Glu152*	Nonsense
7	p.Arg203*	Nonsense
8	p.Arg239Profs*5	Frameshift
9	p.Leu245Glufs*4	Frameshift
10	p.Asp275Metfs*13	Frameshift
11	p.Arg282Glyfs*7	Frameshift
12	c.847-9C>G(283AA)	Splice
13	p.Arg323*	Nonsense
14	p.Arg323Profs*18	Frameshift
15	c.991-2A>G（331AA）	Splice
16	p.Tyr346*	Nonsense
17	c.1085+1G>A（362AA）	Splice
18	c.1166-11_1166-2del	Splice
19	c.1271+1G>A（424AA）	Splice
20	c.1271+4_1271+7delAGTA（424AA）	Splice
21	p.Arg446Cys	Missense

22	p.Arg446His	Missense
23	p.Val456Phe	Missense
24	c.1384_1386+4del（462AA）	Splice
25	c.1386+1G>T	Splice
26	p.Gln474*	Nonsense
27	p.Arg476*	Nonsense
28	p.Glu488_Ala491del	In frame indel
29	p.Glu488_Ala490del	In frame indel
30	p.Ala489_Ala491del	In frame indel
31	p.Ala490_Ala493del	In frame indel
32	p.Ala495Gly	Missense
33	p.Gly504Alafs*72	Frameshift
34	p.Gly506Valfs*71	Frameshift
35	p.Ala533Val	Missense
36	[GRCh37] DEL:chrX-54,820,664-54,839,053(MAGED2:E1-E7 Del)	Exon deletion

37	[GRCh37] DEL:chrX-54,834,585-54,986,301(MAGED2:E2-E13 Del)	Exon deletion
38	MAGED2(E1-E13)	Gene deletion
